# 25.2 UNDERSTANDING WHITE MATTER PATHOLOGY IN SCHIZOPHRENIA USING DIFFUSION TENSOR IMAGING AND MAGNETIC RESONANCE SPECTROSCOPY

**DOI:** 10.1093/schbul/sby014.102

**Published:** 2018-04-01

**Authors:** Adrienne Lahti, Meredith Reid, David White, Nina Kraguljac

**Affiliations:** 1 The University of Alabama at Birmingham; 2 Auburn University

## Abstract

**Background:**

Diffusion tensor imaging (DTI) studies in schizophrenia consistently show global reductions in fractional anisotropy (FA), a putative marker of white matter integrity. Because magnetic resonance spectroscopy (MRS) studies permit for the non-invasive measurements of neurometabolites, such as N-acetylaspartate (NAA), a marker of neuronal integrity, and glutamate, which can be neurotoxic when over-released, this technique provides further understanding of the relationship between white matter microstructure and neuronal function.

**Methods:**

Twenty-nine schizophrenia patients and twenty controls participated in this 3T imaging study where we used DTI and tract-based spatial statistics (TBSS) to assess white matter integrity of the cingulum bundle and MRS to quantify NAA and glutamate in the anterior cingulate cortex (ACC) and hippocampus, i.e. in cortico-limbic regions connected by the cingulum bundle.

**Results:**

We found FA reductions with overlapping radial diffusivity (RD) elevations in patients in multiple tracts, suggesting white matter abnormalities in schizophrenia are driven by loss of myelin integrity. In controls, but not in patients, high hippocampal NAA levels were significantly associated with low RD in the hippocampal part of the cingulum, and low ACC glutamate levels were significantly associated with high FA in the hippocampus part of the cingulum.

**Discussion:**

In conclusion, we demonstrate the potential utility of a multi-modal neuroimaging approach to help further our understanding of the relationship between white matter microstructure and neurochemistry in distinct cortical regions connected by white matter tracts.

